# Cumulative Signal Transmission in Nonlinear Reaction-Diffusion Networks

**DOI:** 10.1371/journal.pone.0062834

**Published:** 2013-05-08

**Authors:** Diego A. Oyarzún, Fernando López-Caamal, Míriam R. García, Richard H. Middleton, Andrea Y. Weiße

**Affiliations:** 1 Centre for Synthetic Biology and Innovation, Department of Bioengineering, Imperial College London, London, United Kingdom; 2 Hamilton Institute, National University of Ireland, Maynooth, County Kildare, Ireland; 3 Centre for Complex Dynamic Systems and Control, The University of Newcastle, Newcastle, New South Wales, Australia; 4 SynthSys–Synthetic and Systems Biology, University of Edinburgh, Edinburgh, United Kingdom; Humboldt University, Germany

## Abstract

Quantifying signal transmission in biochemical systems is key to uncover the mechanisms that cells use to control their responses to environmental stimuli. In this work we use the time-integral of chemical species as a measure of a network’s ability to cumulatively transmit signals encoded in spatiotemporal concentrations. We identify a class of nonlinear reaction-diffusion networks in which the time-integrals of some species can be computed analytically. The derived time-integrals do not require knowledge of the solution of the reaction-diffusion equation, and we provide a simple graphical test to check if a given network belongs to the proposed class. The formulae for the time-integrals reveal how the kinetic parameters shape signal transmission in a network under spatiotemporal stimuli. We use these to show that a canonical complex-formation mechanism behaves as a spatial low-pass filter, the bandwidth of which is inversely proportional to the diffusion length of the ligand.

## Introduction

Cell survival hinges on the ability to respond to extracellular stimuli and self-regulate in a changing environment. Intracellular dynamics are controlled by intricate arrays of biochemical networks, and in particular, the spatiotemporal dynamics of species concentrations are key to a number of processes, including cell signalling [1], pattern formation [2] and morphogenesis [3]. Quantifying the signal transmission properties of a network is key to understand how its connectivity and parameters shape the conversion of signalling cues into cellular responses, as well as the detection of intervention points for engineering or therapeutic applications.

Our goal in this paper is to provide tools for the mathematical quantification of signal transmission in biochemical networks. We use the time-integral of species concentrations as a proxy for the ability of a network to transmit input cues. It represents the cumulative effect of external stimuli on the chemical species and has been used to discover an input amplification phenomenon in the MAPK pathway [4], and to study the activation of cell membrane receptor such as the epidermal growth factor and the erythropoietin receptors [5], [6].

We focus on networks of biochemical reactions subject to molecular diffusion and spatiotemporal stimuli. We aim at obtaining *exact formulae* for the time-integrals of species concentrations. An analytic approach can reveal structural properties of the model under consideration, as opposed to simulation-based studies where it is unclear if predictions are rather a consequence of the particular parameter values examined. In the case of diffusionless systems, the work in [7] provided exact expressions for the 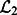 norm of a class of signalling cascades. However, similar results for reaction-diffusion systems remain elusive, owing to the fact that the vast majority of nonlinear reaction-diffusion systems are analytically intractable.

A complete solution to this problem, for any reaction-diffusion network, may require analytic solutions of the reaction-diffusion partial differential equation (PDE). We have previously identified a class of nonlinear networks in which the time-integrals of some species can be computed as a series [8]. Here we build on these results and show that in this class the time-integrals satisfy a linear inhomogeneous differential equation. Solving the derived equation leads to analytic expressions for the time-integrals without knowing the solution of the nonlinear PDE. We further provide a graphical characterization of the class of networks in terms of the Species-Reaction graph [9]. This provides a simple test to determine if a given network belongs to the derived class and to explore other network topologies that are amenable to our analysis. Applying our results to a complex-formation mechanism with sigmoidal kinetics, we show that it behaves as a spatial low-pass filter and that the temporal response can display a “waterbed effect” whereby concentrations ripple around their steady state and lead to a nil time-integral.

## Results and Discussion

### Exact Computation of the Time-integrals

We consider networks composed of 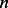 species interacting through 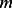 reactions:
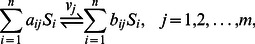
(1)where 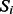 is the 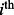 reactant or product for the 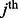 reaction. The numbers 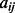 and 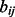 denote the stoichiometric coefficients of the corresponding species. The reaction-diffusion model for the network is
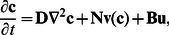
(2)where 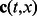 is the vector of 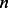 species concentrations, 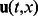 is a vector of 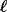 influx/efflux rates accounting for environmental stimuli, and 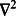 is the Laplacian operator (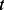 and 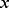 denote time and space coordinates, respectively). The nonlinear vector function 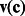 contains the 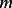 reaction rates, whereas the matrices 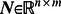 (with 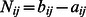), 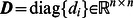 and 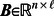 describe the stoichiometry, diffusion coefficients, and which species are subject to external stimuli.

We focus on the response of the reaction-diffusion network to an initial spatial perturbation 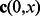 and a transient spatiotemporal stimulus 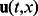 such that 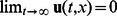 and 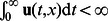. For simplicity here we will focus on a 1D domain 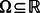 with the same boundary conditions for all species. Once the effect of the stimulus 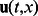 has vanished, we assume that the network reaches a unique homogeneous equilibrium 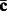.

One way of quantifying the network response is by means of the time-integral:
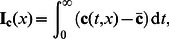
(3)which is finite provided that the equilibrium is exponentially stable. We relabel and partition the species and reaction rate vectors as follows:

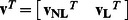
, where 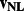 contains 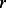 nonlinear rates and 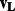 contains the remaining 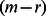 affine rates,
- and 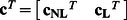, where 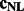 contains the 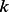 species that react only in nonlinear reactions, 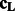 includes the remaining 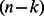 species.

The affine reaction rates contain a combination of zeroth and first order terms of the form 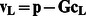, with 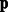 a vector of constant production rates and 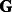 is a matrix of first order kinetic constants. The nonlinear rates typically model saturable binding kinetics such as Michaelis-Menten or Hill kinetics [10], together with linear dissociation (note that in our notation reversible reactions are taken as a single rate). If 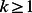 we can find a labelling for the reaction rates so that the stoichiometric matrix has a block-triangular form
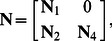
(4)with 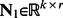, 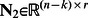 and 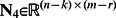.

We found that under the following conditions (see Analysis section for details):

**C1** the species in 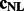 do not diffuse, and

**C2** the number of species in 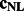 is equal to the number of nonlinear reactions (i.e. 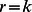), the time-integral of 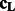 satisfies the differential equation
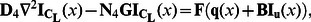
(5)where 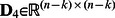 is the diffusion matrix of 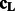, 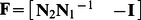, 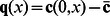, and 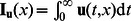. The solution of (5) must satisfy boundary conditions consistent with those of the reaction-diffusion PDE. Equation (5) is an inhomogeneous linear differential equation with constant coefficients, and therefore depending on the spatial profile of the stimuli 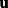 and initial condition 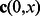, it may be possible to obtain a closed-form solution for the time-integral 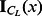.

In the general case when a closed-form solution is not available, equation (5) can be solved by projecting the solution on an orthonormal basis for the spatial domain 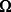. To this end, we write 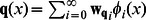 and 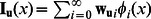, where 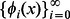 is a complete orthonormal basis of 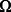. We choose the basis as orthonormal eigenfunctions of the Laplacian, i.e. 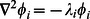 subject to the boundary conditions [11]. The time-integrals are then 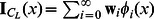 with coefficients
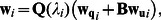
(6)and 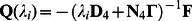. The derived series is *exact* and we can use it to compute the time-integrals of 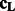 without knowing the solution of the nonlinear PDE. Most importantly, the series coefficients 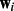 are explicitly given in terms of the geometry and boundary conditions (comprised in the eigenvalues 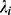), the initial condition and the equilibrium (comprised in the function 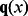), and the total concentration supplied to and consumed from the network (comprised in the integral of 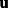). Note that these coefficients can also be obtained by linearizing the PDE in (2), but such an approach provides no guarantee of the exactness of the solution. Linearized solutions neglect the nonlinear terms in the PDE, and therefore they are valid only for small perturbations around the equilibrium. In our case, conditions **C1** and **C2** guarantee that the derived time-integral is exact, defining a class of nonlinear networks for which the time-integral can be computed analytically for small or large perturbations.

### Graph Interpretation of the Network Conditions

**Figure 1:**
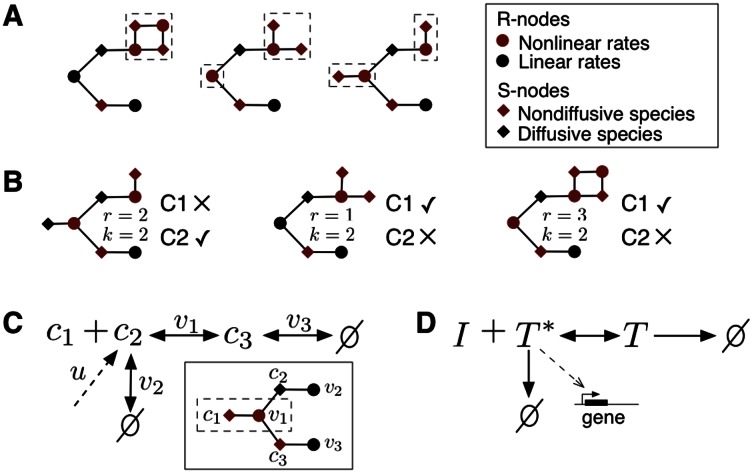
Conditions for the computation of the species’ time-integrals in terms of the Species-Reaction graph. The conditions amount to the graph having a (possibly disjoint) subgraph containing every red R-node with all their adjacent red S-nodes not linked with any black R-nodes; in this subgraph, the number of red R-nodes and red S-nodes must be the same. (**A**) Networks with two nonlinear reactions (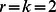) satisfying the conditions. The red subgraphs are marked with dashed boxes. (**B**) Networks that do not satisfy the conditions. (**C**) A generic complex-formation mechanism satisfying the conditions. A spatially-fixed molecule 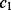 binds a diffusible ligand 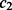 to form a complex 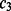. Species 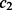 and 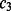 are synthesized at a constant rate and linearly degraded. External stimuli of ligand can be modeled via a spatiotemporal influx 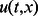. The Species-Reaction graph is shown in the inset. (**D**) Genetic regulation via protein sequestration [12] is an instance of the mechanism in **C**. A nondiffusive inhibitor 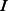 sequesters a transcriptional activator 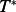 to form an inactive complex 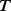, causing the downregulation of gene expression.

Conditions **C1** and **C2** are structural (hence independent of the functional form of the nonlinearities) and can be interpreted in terms of a graph. We use the Species-Reaction graph (Fig. 1 A), composed of two sets of nodes [9]: species nodes, denoted as S-nodes, and reaction nodes, denoted as R-nodes. The graph is bipartite–so that reaction nodes only link to species nodes, and *vice versa*–and is defined as follows: an S-node 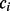 is connected to an R-node 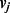 if 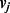 depends on 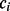 (i.e. 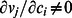). As shown in Fig. 1 A, we color the S-nodes black (red) if they are diffusive (nondiffusive) species, and the R-nodes black (red) if they correspond to linear (nonlinear) reaction rates. With these definitions, conditions **C1** and **C2** amount to:

C^*^ all S-nodes that are not connected to black R-nodes are red, and their number equals the number of red R-nodes.

As illustrated by the examples in Fig. 1 A, under condition **C
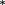
** the network graph contains a red subgraph that corresponds to the nonlinear and nondiffusive portion of the network that is not directly connected to any linear reactions (see Fig. 1 B for examples where the conditions are not met). Under these conditions we can use our formula to compute the time-integrals of all the species outside the subgraph.

Condition **C1** is generally valid for species with large molecular weight or that are spatially fixed, such as membrane-bound receptors or molecules anchored to the cytoskeleton. Condition **C2** is more restrictive because it requires the nonlinear and nondiffusive part of the network to have as many reactions as species. A particularly relevant system that meets the conditions is the generic complex-formation network in Fig. 1 C, where a diffusible ligand forms a complex with an immobile molecule. This mechanism can be found, for example, in ligand-receptor interactions [5] and protein sequestration [12]. In the latter case, if the sequestered protein is a transcription factor for a specific gene (Fig. 1 D), cells can use sequestration to downregulate gene expression in response to intracellular signals.

If we write the perturbation (both the initial spatial perturbation 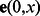 and the spatiotemporal stimulus 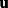) in the basis of 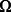, the coefficients 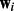 in (6) can be seen as the product of those of the perturbation and the coefficients comprised in the matrix 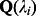. For a number of spatial geometries, the eigenfunctions of the Laplacian are typically sine and/or cosine functions [13], and thus the resulting time-integral can be understood as a filtered version of the perturbations. The form of the coefficients in (6) also indicates that if a column of 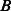 is orthogonal to a row of 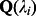 for all 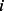, then the corresponding input will generate a nil time-integral. We can use this property to detect input channels that generate a “waterbed effect”, where concentrations ripple around the equilibrium and lead to a nil time-integral.

### Complex-formation Network under Spatiotemporal Stimuli

To illustrate the utility of our approach we use it in the complex-formation network in Fig. 1 C. The reaction-diffusion PDE for this network in the domain 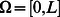 is
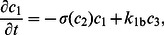
(7)

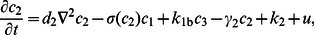
(8)

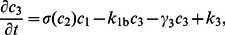
(9)where 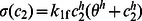, 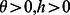 is a sigmoidal nonlinearity describing the binding between 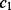 and 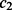, 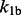 is their dissociation rate constant, 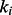 are the synthesis rates, and 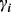 are the degradation rate constants.

We partition the rate vector into its nonlinear and linear components, i.e. 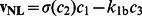 and 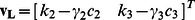, and the species as 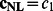 and 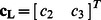. For homogeneous Neumann boundary conditions (i.e. 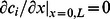 for 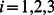), it can be shown that the network has a unique homogeneous equilibrium point at 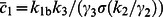, 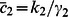 and 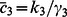. The blocks of the stoichiometric matrix are 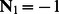, 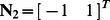 and 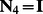, whereas the matrix of first order kinetic constants is 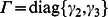 and 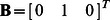. The eigenfunctions of the Laplacian in 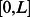 are 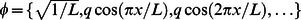, with 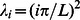 and 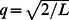. From (5) we can obtain an ordinary differential equation for the integral of 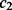 and an explicit expression for the one of 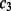 (note that 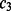 does not diffuse):
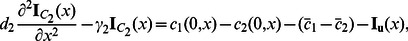
(10)

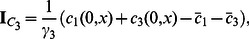
(11)with 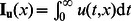 and subject to boundary conditions 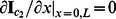. We next consider the network response to two types of perturbations: a purely spatial perturbation and a spatiotemporal influx of ligand.

### Spatial Perturbation

**Figure 2:**
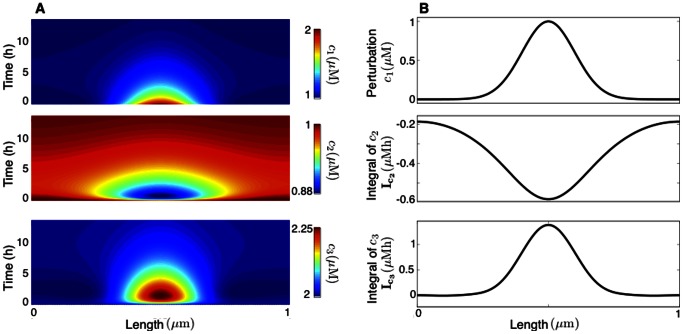
Response of the network in Fig. 1 C to a spatial perturbation in 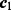. (**A**) Species concentrations. (**B**) Gaussian perturbation 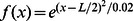 and the time-integrals of species 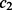 and 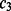. The parameters are 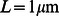, 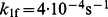, 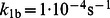, 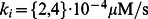, 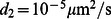, 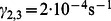, 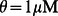, and 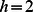.

We first consider the case of an initial spatial perturbation in 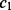 of the form 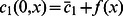, where 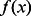 is the spatial profile of the perturbation and there is no stimulus (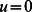). The other species are initially at equilibrium, i.e. 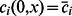 for 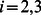, so that 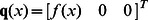 in (5). We can write the perturbation in the basis as
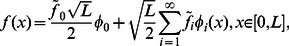
(12)with 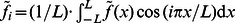 and 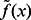 is the even 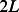-periodic extension of 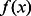. Note that the expansion in (12) converges to 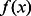 only in the domain 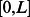. Using (6) we get the time-integrals
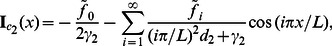
(13)and 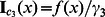. In Fig. 2 we show the response of the network to a Gaussian spatial perturbation under zero flux boundary conditions, together with the time-integrals 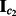 and 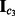.

The coefficients 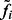 describe the frequency content of the perturbation (plus spurious harmonics arising from the periodic extension). The expressions for 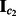 and 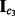 therefore indicate that the time-integral of the diffusible species is a filtered version of the spatial perturbation, and this filtering effect disappears in the case of the immobile species. Diffusion of 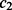 acts as a spatial filter with a low-pass characteristic [14], and the magnitude of its frequency response is 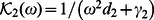, representing the attenuation factor for a spatial harmonic of frequency 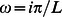.

An important parameter of reaction-diffusion systems is the *diffusion length* 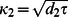, where 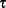 is the species half-life. It represents the distance a molecule typically diffuses over its lifetime and determines the length scale of the diffusion process [15]. The cutoff frequency of the spatial filter (the frequency above which harmonics are attenuated by at least 50%) is 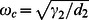, thus inversely proportional to the diffusion length. This indicates that in the mechanism of Fig. 1 C, ligands with a short diffusion length behave as high-bandwidth filters that encode a rich harmonic content in their time-integral.

Conversely, molecules with long diffusion lengths may suppress all harmonics and lead to a spatially homogeneous time-integral. This attenuation can be drastic, for example, in cytosolic proteins of *S. cerevisiae*, whose mean diffusion coefficient and diffusion length have been recently estimated at 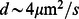 and 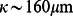 (based on 1400 proteins [16]). Considering the typical diameter of *S. cerevisiae* 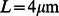, we conclude from 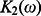 that the first harmonic of the perturbation is subject to 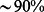 attenuation, and any higher harmonic will be attenuated by a larger factor. In these cases, we suggest that information encoded in the perturbation may be more faithfully transmitted through transient-dependent features, such as the peak value or the response time of the species concentrations. These quantities have been extensively used in diffusionless models for biochemical networks [4], [6], but remain largely unexplored when molecular diffusion is not negligible.

### Spatiotemporal Influx

**Figure 3:**
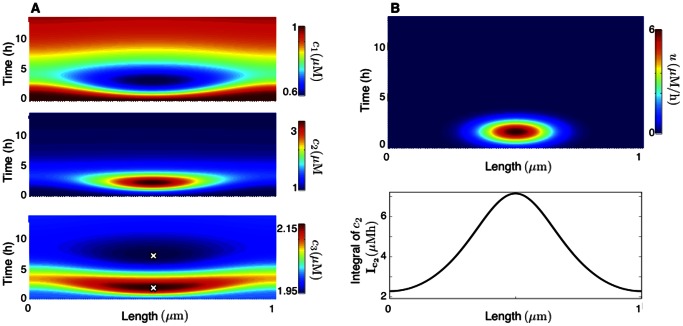
Response of the network in Fig. 1 C to a spatiotemporal influx of ligand 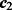. (**A**) Species concentrations; the white crosses mark the peak and valley of 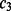. (**B**) Gaussian influx 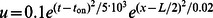 and time-integral of the ligand. Parameter values are the same as in Fig. 2.

We now consider that all species are initially in equilibrium, i.e. 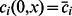 for 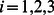, and a spatiotemporal influx of ligand 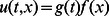. In this case we have 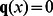 and from (6) we get
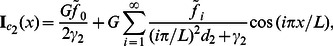
(14)and 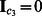 with 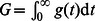. In Fig. 3 we show the network response to a spatiotemporal Gaussian pulse of ligand influx under zero flux boundary conditions, together with the time-integral 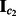.

The nil time-integral of 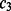 indicates that the areas above and below the equilibrium cancel out, leading to a waterbed effect. In Fig. 3 A we effectively observe that 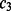 peaks and then undershoots below its equilibrium, subsequently recovering back to its pre-stimulus level. The time-integral of 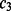 is zero for all points in space and reveals a fundamental tradeoff in the response: its peak can be amplified only at the expense of a deeper valley under the equilibrium. This type of tradeoff arises from the network structure and is independent of the parameter values, emphasizing the role of model analysis in applications that require a precise control of biological responses, such as the delivery of growth factors in tissue engineering [17] or the control of pattern formation [18].

### Concluding Remarks

In this work we discussed the analytic computation of time-integrals in nonlinear reaction-diffusion systems. We found conditions under which the time-integrals of some species satisfy a linear differential equation, the solution of which can be written as a function of the kinetic parameters, the geometry and the spatiotemporal stimuli. The derived conditions represent constraints on the interaction topology between the nonlinear rates and nondiffusive species. They depend only on the network topology and are independent of the functional form of the kinetic nonlinearities.

We recast the conditions in terms of a graph that provides a simple test to check their validity in any given network and a means to find other topologies where our analysis can be applied. The graph interpretation suggests the conditions are well suited for systems with a small number of nonlinear reactions and whose diffusive reactants appear also in first order reactions. This narrows down the class of networks amenable to our result, albeit this is not surprising since analytic solutions for nonlinear PDEs are rarely available. Moreover, typical reaction-diffusion models have a small number of species and reactions, as their analysis can become increasingly complex in high dimensions (even in low dimensional cases they can display a wide range of complex dynamics [19]). In those models that do satisfy the required conditions, the analytic relationship between the time-integrals and the model parameters can reveal substantial insights into the network dynamics.

We showed that a model for protein sequestration–a ubiquitous mechanism in cell regulation–can be readily analyzed with our theory. Other relevant mechanisms amenable to our approach include membrane receptor systems [20] and calcium sequestration by immobile buffers [21]. We illustrated our results in a canonical complex-formation mechanism with sigmoidal binding kinetics. This is a non-trivial and biologically relevant system where the reaction-diffusion PDE has no known analytic solution. We showed that this mechanism behaves as a low-pass filter and displays a waterbed effect [22] in the dynamic response of the complex for all parameter values and a wide range of spatiotemporal stimuli. Analytic approaches such as the one presented here can shed light on the mechanisms by which living cells modulate their responses to environmental cues. This can ultimately lead to the identification of key control parameters that can be targeted to modify cellular responses, for example, with the use of therapeutic drugs.

### Analysis

Here we show how to obtain the differential equation for the time-integrals in (5), and the series coefficients in (6). With the chosen partitions for the reaction rates and species concentrations, we can write
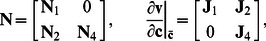
(15)with 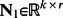, 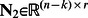, 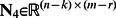, 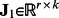, 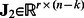, and 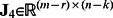. Exponential stability of the equilibrium 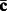 implies that the matrix
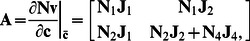
(16)is invertible [23], which means that 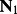 is full column rank (note that otherwise, if there exists a vector 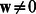 such that 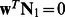, then 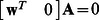, which implies that 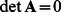 and contradicts the invertibility of 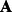). In addition, by Condition C2 the matrix 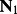 is square and therefore 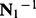 is well defined. By Condition C1 the reaction-diffusion PDE in (2) can be written as
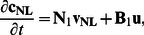
(17)

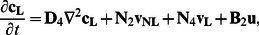
(18)where 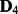 is the diffusion matrix of 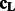 and 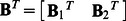. Note that setting (17) and (18) to zero for 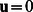, we get that any homogeneous equilibrium 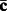 satisfies 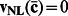 (because 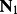 is a nonsingular matrix) and 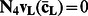. Using the form of the affine rates 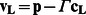, we conclude that the homogeneous equilibrium for 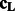 satisfies 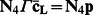. From (17) we can solve for 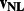 to get 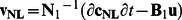, which after substituting in (18) and rearranging terms yields
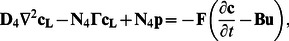
(19)with 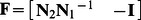. Using the relation 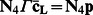, the equation in (19) can be rewritten as



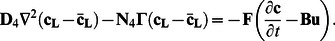
(20)The differential equation for 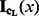 in (5) can be obtained by integrating (20) from 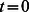 to 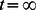. To get the coefficients for the series in (6), we substitute the series for 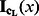, 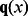 and 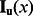 in (5):
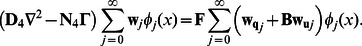
(21)


Since the basis satisfies the eigenvalue problem 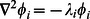, from (21) we get
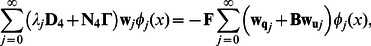
(22)


Postmultiplying (22) by 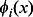 and integrating over the spatial domain leads to one equation for each coefficient 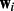


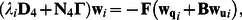
(23)for 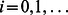. To obtain (23) we used the orthonormality of the basis (i.e. 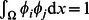 for 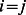 and zero otherwise). The final expression for the coefficients in (6) can be obtained directly from (23).
